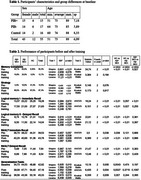# Behavioral Outcomes of Cognitive Training in Patients with Amnestic Mild Cognitive Impairment, With and Without Amyloid Pathology, and a Control Group

**DOI:** 10.1002/alz70857_107334

**Published:** 2025-12-26

**Authors:** Nadia A Bossa, Eliane C Miotto, Geraldo Busatto Filho, Ricardo Nitrini, Sonia Brucki

**Affiliations:** ^1^ University of São Paulo, São Paulo, São Paulo, Brazil; ^2^ University of São Paulo, São Paulo, SP, Brazil; ^3^ Department of Neurology, Faculdade de Medicina FMUSP, Universidade de Sao Paulo, Sao Paulo, Sao Paulo, Brazil; ^4^ University of São Paulo Medical School, São Paulo, Brazil

## Abstract

**Background:**

This study aimed to evaluate the impact of image‐based unimodal cognitive training (CT) on cognitive function in Aβ+ and Aβ‐ MCI patients compared to healthy controls (HC).

**Method:**

Fifty‐five older adults participated: 19 Aβ+ MCI, 16 Aβ‐ MCI, and 8 HC. All underwent neuropsychological assessment before and after CT. The assessment included a Memory Complaints and Strategy Use Questionnaire, which evaluated subjective memory difficulties and the spontaneous use of mnemonic strategies before and after training; performance in a memory task (Generalization Tasks) with and without images (Baseline, Post‐Training, and 9‐month Follow‐up); Logical Memory (LM 1 – Immediate Recall, LM 2 – Delayed Recall); Rey Auditory Verbal Learning Test (RAVLT A1‐A5 – Immediate Recall, RAVLT A7 – Delayed Recall). Statistical analyses included ANOVA or Kruskal‐Wallis, depending on normality (Shapiro‐Wilk) and homogeneity of variances (Levene). For group comparisons, Dunn's post hoc test was applied following Kruskal‐Wallis, while Tukey's test was used for ANOVA.

**Result:**

Memory Complaints: Before training, 94.74% of Aβ+ and 85% of Aβ‐ reported memory complaints, compared to 6.5% in HC. After training, complaints reduced to 15%, 20%, and 0%, respectively. Use of Strategies: Initially, only 5.26% of Aβ+ and 10% of Aβ‐ used memory strategies, compared to 31.25% in HC. After training, strategy use increased in the MCI groups. Memory Performance: Immediate Recall (LM 1 and RAVLT A1‐A5): Pre‐training, HC performed significantly better than Aβ+ (*p* < 0.05). After training, all three groups improved, reducing the gap between Aβ+ and Aβ‐ in relation to HC; Delayed Recall (LM 2 and RAVLT A7): HC performed significantly better than Aβ+ and Aβ‐ (*p* < 0.01 pre and post‐training). Both MCI groups improved, but differences remained; and Generalization Tasks: At baseline, HC had significantly better scores (*p* < 0.0061). Post‐training, HC continued to outperform Aβ+ and Aβ‐ (*p* < 0.01).

**Conclusion:**

CT improved memory, strategy use, and generalization in the Aβ+, Aβ‐, and HC groups. Regarding immediate and delayed memory, statistical analysis showed that all groups improved post‐training, with the Aβ‐ group benefiting the most.